# T2 mapping and T2* imaging in heart failure

**DOI:** 10.1007/s10741-017-9616-5

**Published:** 2017-05-11

**Authors:** A.S. Lota, P.D. Gatehouse, R.H. Mohiaddin

**Affiliations:** Cardiovascular Biomedical Research Unit, Royal Brompton Hospital, National Heart and Lung Institute, Imperial College, Sydney Street, London, SW3 6NP UK

**Keywords:** T2 mapping, Inflammatory cardiomyopathy, Iron overload, Myocarditis, Sarcoidosis, Transplant rejection

## Abstract

Cardiovascular magnetic resonance (CMR) is a versatile imaging modality that enables aetiological assessment and provides additional information to that of standard echocardiography in a significant proportion of patients with heart failure. In addition to highly accurate and reproducible assessment of ventricular volumes and replacement fibrosis, multiparametric mapping techniques have rapidly evolved to further expand the diagnostic and prognostic applications in various conditions ranging from acute inflammatory and ischaemic cardiomyopathy, to cardiac involvement in systemic diseases such as sarcoidosis and iron overload cardiomyopathy. In this review, we discuss the established role of T2* imaging and rapidly evolving clinical applications of myocardial T2 mapping as quantitative adjuncts to established qualitative imaging techniques.

## Introduction

Cardiovascular magnetic resonance (CMR) has developed over several decades from an ancillary research tool to an evidence-based imaging modality that remains not only the gold standard assessment of cardiac morphology and ventricular function, but also has the additional benefit of in vivo tissue characterisation. Whilst limited in acutely decompensated states where patients may not tolerate lying flat, CMR plays a central role in the diagnostic evaluation and risk stratification of patients with heart failure [1, 2].

### Conventional CMR imaging

Biological systems of magnetic resonance imaging measure the energy released by protons during the relaxation phase as they recover back towards equilibrium after a radio-frequency pulse. The relaxation consists of two types: *recovery* of the longitudinal component of magnetisation (the T1 relaxation time) towards equilibrium (‘spin-lattice’ coupling transferring energy out of the nuclear magnetisation) and the *decay* of the measurable transverse magnetisation (the T2 relaxation time) by *irreversible* effects (as opposed to others described later), known as spin-spin coupling. The T1 and T2 values vary depending on the composition of different biological tissues, primarily increased by greater water content, and these fundamental differences form the basis of intrinsic contrast used to generate images. Notably, cell cytoplasm is usually more laden with large molecules and tends to show shorter T2 than the usually purer interstitial fluid, although this is usually combined within a single MRI voxel as a complicated average [3, 4].

Conventional imaging is reliant upon qualitative visual analysis of signal intensity on the acquired images, which may be altered by adjusting the pulse sequence for T1 and T2 weighting. For example, T2-weighted imaging has an established role in depicting myocardial oedema due to the effect of increased interstitial free water on lengthening T2 relaxation times with particular relevance to inflammatory conditions, such as a myocarditis and sarcoidosis, and acute ischaemic injury. Similarly, the presence of increased iron reduces T2 and T1 by local magnetic field distortion. Myocardial image contrast can also be extrinsically modified through the intravenous administration of gadolinium contrast agent, which usually remains extracellular, where T1-weighted imaging shows areas of injured myocardium with expanded extracellular space due to shortened T1 recovery times. These findings have been confirmed by extensive histological validation in past decades, with accumulating clinical outcome-based studies also confirming prognostic significance, for example, in non-ischaemic dilated cardiomyopathy [5].

However, signal intensity ratios in these conventional CMR imaging sequences are displayed on an arbitrary grey scale and therefore are not suited to quantitative measurement or comparison between patients and serial examinations. Subjective visual analysis susceptible to interobserver variation represents the main limitation of conventional CMR imaging.

### T2-STIR

T2-weighted imaging shows increased myocardial signal from myocardial oedema based on the prolongation of the T2 relaxation caused by the accumulation of interstitial water. This was first demonstrated in 1983 in a canine model of acute myocardial infarction [6]. T2 relaxation refers to the natural interactions causing irreversible dephasing of transverse magnetisation at atomic or molecular scale. Spin-echo sequences are used with a re-focusing (180°) radiofrequency pulse to re-phase reversible loss of transverse magnetisation due to local magnetic field inhomogeneity at larger scales, which can be considered stationary over the relevant duration involved during measurement. Signal from fat and the blood pool is suppressed to improve image quality. Sequences typically use a short tau inversion recovery (STIR) nulled to suppress the shorter T1 of fat with a double-inversion-recovery method aiming to suppress blood signal, overall known as ‘triple-inversion recovery,’ in preparation for a fast spin-echo sequence with T2 image contrast weighting identified loosely hereafter as T2-STIR. In this way, pronounced contrast is created between bright oedema (longer T2) and hypointense normal myocardium (shorter normal T2).

**Fig. 1:**
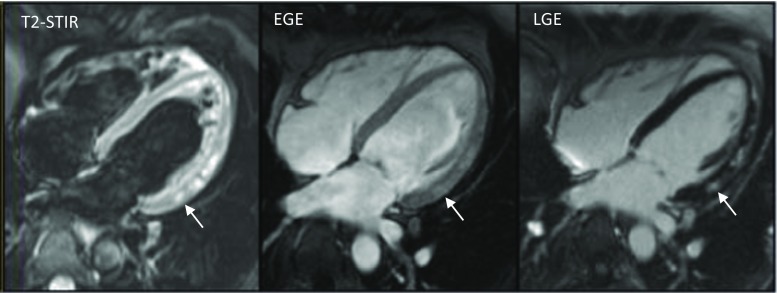
Standard Lake Louise Criteria for acute myocarditis showing focal regions of myocardial oedema on T2-STIR, reactive hyperaemia on early gadolinium enhancement (EGE) and myocyte necrosis/fibrosis on late enhancement (LGE) in the inferolateral wall (*arrowed*)

Preclinical and human studies have demonstrated a range of clinical applications for this technique, for example, in acute myocardial infarction and acute myocarditis (Fig. 1) [7]. However, limitations are well known to include low signal-to-noise ratio, loss of signal due to cardiac motion (not only the spin-echo method but also the complex triple-IR preparation sequence), imperfect blood suppression in areas of slow blood flow and subjective visual interpretation. Whilst focal T2 increases may be easily visualised as image resolution of T2-STIR is finer than that of T2 mapping, larger regions are more challenging as they are easily confounded by myocardial signal darkening linked to motion and incorporate many other uncontrolled factors in MRI signal brightness—as yet, no standardised calibration of MRI magnitude values is routinely possible.

### T2-STAR

T2* relaxation represents the inherent decay of transverse magnetisation caused by a combination of spin-spin relaxation (T2) and magnetic field inhomogeneity (T2’), which in modern systems is dominated by tissue diamagnetic susceptibility-induced field distortions from the presence of paramagnetic materials such as iron. Gradient echo (GRE) sequences are optimised for T2* weighting by using a low flip angle, long echo time (usually a series of echo times (TE) to support T2* calculation) and, by definition, will not include a refocusing pulse to correct dephasing due to magnetic field inhomogeneity. A normal mean T2* value of above 40 ms has been widely reported in healthy volunteers [8]. The presence of increased tissue iron results in faster T2* relaxation due to susceptibility-induced field distortions, which reduces signal intensity more rapidly as TE increases—this can be visualised as darkening of myocardial (and liver) tissue proportional to the iron concentration.

### T2 mapping

**Fig. 2:**
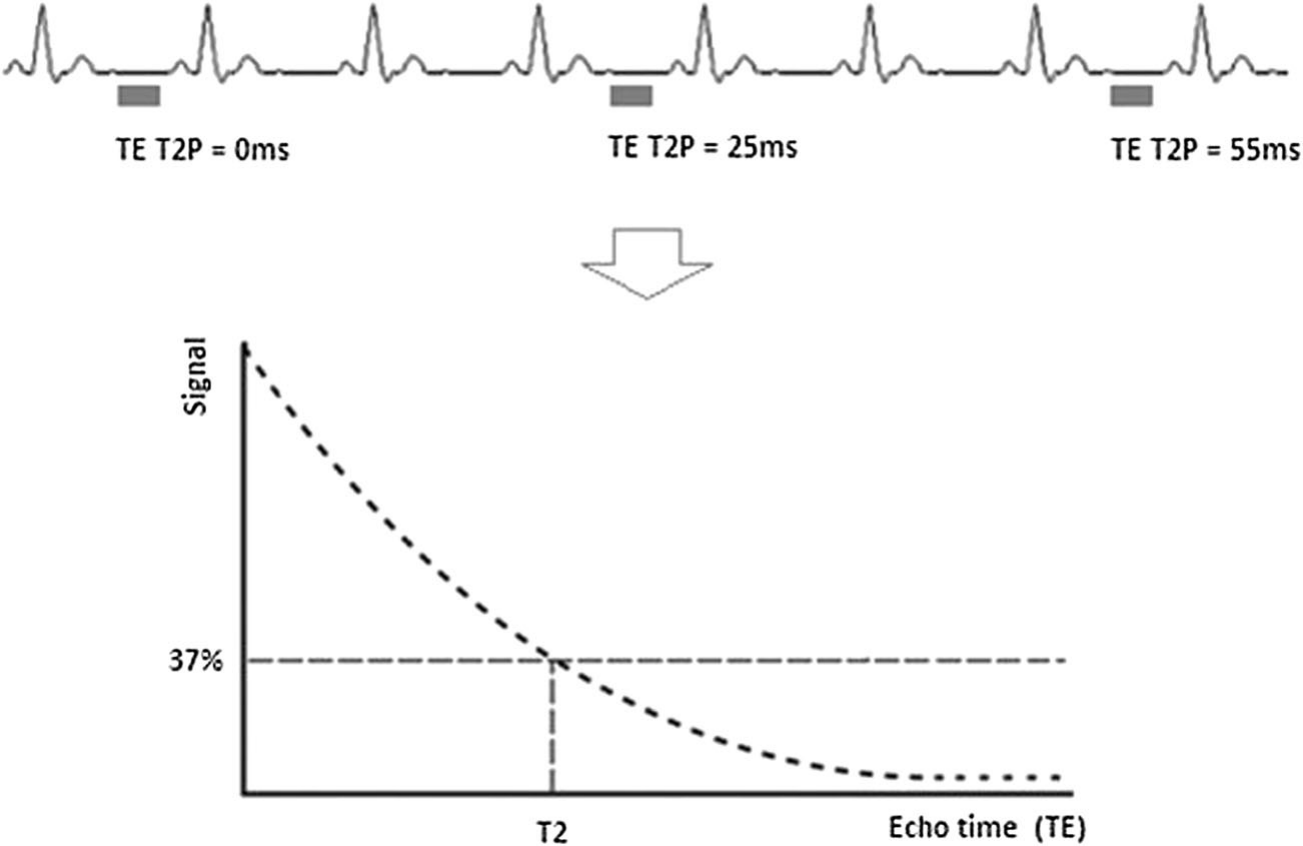
Principles of T2 mapping with different T2 preparatory durations with a long repetition time between the used cardiac cycles, crucial to allow as complete T1 recovery as possible, followed by reconstruction of the transverse relaxation curve in each pixel assuming satisfactory registration. T2 is defined as the time in milliseconds by which the transverse magnetisation has decayed to 37% of the original value. Many distorting factors are not illustrated, and many T2 mapping sequences ‘fill the gaps’ with gradient activity without RF, so that the patient does not consider the scan complete and start breathing too early

T2 mapping, or T2 transverse relaxation time mapping, is a technique used to construct a ‘parametric image’ or ‘map’ in which the intensity of each voxel is the output of a calculation performed independently at each corresponding spatial pixel from a series of input images. The map value reflects the calculated T2 relaxation time at each pixel. T2 maps can be analysed visually on a grey (or colour) scale but can also be analysed quantitatively by defining regions of interest relevant to the particular pathology being studied. Various different sequences have been used for T2 mapping. In principle, at least three separate single-shot images are acquired at increasing T2 preparation times to construct a transverse relaxation curve from these separate TE (Fig. 2) [9]. A long repetition time of two to four RR intervals is used to achieve maximal T1 longitudinal recovery, which otherwise is capable of distorting the calculated T2 presented in the map without any warning—caveat emptor certainly applies [10]. Motion correction algorithms are often used given that at least three T2-weighted images are acquired over multiple heart beats during a single breath-hold. Parametric mapping can be performed in any cardiac slice and position, but most commonly, data is acquired on a short-axis view at the basal and mid-ventricular level. Long-axis views may also be acquired because short-axis slices at the apical level are prone to partial volume effects [11]. Other limitations include the need for increasing the number of RR intervals between each acquisition at faster heart rates to allow complete T1 relaxation [12].

T2 mapping gives access to global T2 changes as well as to nominally measured values for T2 rather than an uncalibrated T2-STIR report. However, the measurement is subject to sequence parameters without a standard, requiring care against changes. For example, some but not all protocols include a T2 preparation image at 0 ms to avoid potential errors invoked by the T2 preparation. As mentioned earlier, the spatial imaging resolution of T2-STIR is finer than T2 mapping and this could be important for detailed focal disease visualisation on T2 maps. In this review, we will evaluate the expanding role of T2 mapping in the assessment of patients with heart failure, which we predict will follow a similar trajectory as T2* imaging for iron overload, from a specialist research technique to a clinically validated tool in widespread general use.

## T2* and iron overload cardiomyopathy

Heart failure due to iron overload is the most common cause of death in patients with thalassaemia major worldwide [13]. However, this form of cardiomyopathy is reversible with prompt initiation of chelation therapy. Serum ferritin does not provide a reliable indication of cardiac iron and conventional imaging techniques to monitor ejection fraction are limited by the late onset of ventricular dysfunction, which only becomes apparent after significant iron deposition has occurred [14]. High cardiac output states seen in chronic anaemia can also mask ventricular dysfunction in some patients. Invasive approaches include endomyocardial biopsy, but this technique is limited by sampling error and is not ideal for serial monitoring, whilst hepatic iron concentration does not give a reliable indication of cardiac iron in cross-sectional studies [15].

**Fig. 3:**
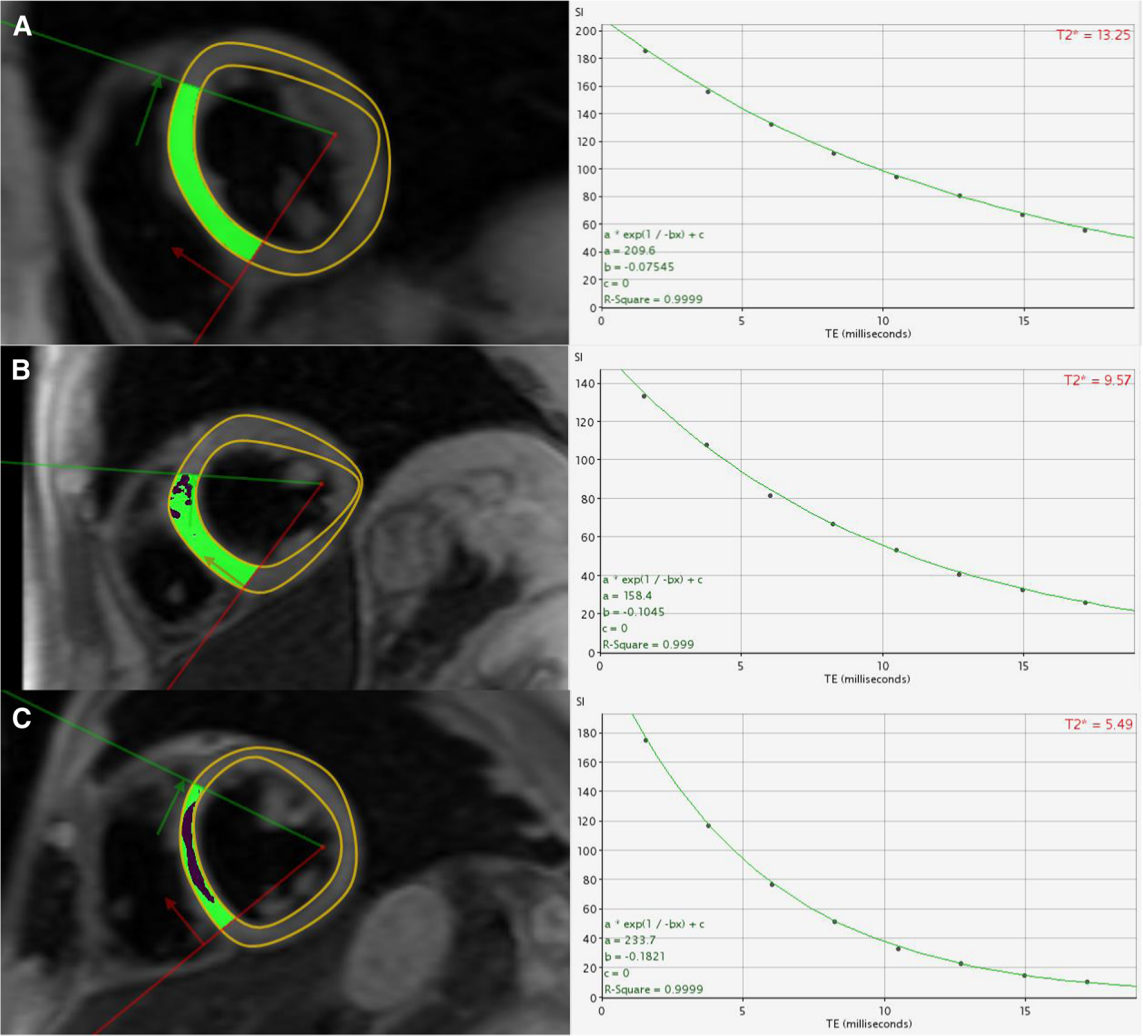
T2* transverse relaxation curves in three separate patients with mild >14 ms (**a**), moderate 10–14 ms (**b**) and severe <6 ms (**c**) iron overload. Black blood T2* imaging is used rather than white blood due to superior reproducibility and reduced imaging artefact [61]

Iron assessment by T2* relies on the measurement of T2* relaxation from GRE sequences. When the storage capacity of ferritin is exceeded, iron is deposited in myocardial and hepatic tissue as particulate haemosiderin, which is a form of ferrihydrite (hydrated iron oxide). This disrupts the local magnetic field homogeneity shortening T2* values with progressive ventricular dysfunction occurring below a T2* threshold of 20 ms [15]. T2* values in the mid-septum have been calibrated to myocardial tissue iron levels in [Fe] milligrams per gram dry weight and indicate a strong inverse linear relationship [8]. Based on a study of 652 thalassaemia patients, T2* was <10 ms in 98% of patients who developed heart failure with the likelihood of left ventricular dysfunction increasing progressively with lower T2* values: 14% at 8–10 ms, 30% at 6–8 ms and 47% at <6 ms (Fig. 3) [16]. In addition, T2* <20 ms conferred an overall relative risk for arrhythmia (atrial and ventricular) of 4.6 (95% confidence interval, 2.66 to 7.95), which also increased using similar increments in T2* [16].

T2* has also effectively been used to monitor treatment response and assess the efficacy of iron chelation in a number of randomised trials [17]. Improvement in myocardial T2* and left ventricular ejection fraction has consistently been observed with oral deferiprone [18] and intravenous desferrioxamine chelation therapy [19]. In cases of severe cardiac iron loading, or when LV function is impaired, a combined treatment approach may also be used [20].

T2* monitoring is now internationally recommended based on these studies in the annual monitoring of transfusion-dependent patients at risk of developing myocardial iron loading [21]. In this way, T2* imaging of cardiac iron loading has progressed from a research technique to a clinically validated tool and has transformed clinical outcomes in β-thalassaemia major [22].

## T2 mapping and acute inflammatory cardiomyopathy

Acute inflammatory cardiomyopathy is a clinical entity that generally requires endomyocardial biopsy to allow assessment of disease activity, characterised by the presence of myocardial oedema arising from inflammation-related increased capillary leakage. Whilst T2 mapping techniques have not received the same focus as T1 mapping in heart failure [23], a growing evidence base indicates that T2 mapping may potentially provide a valuable clinical tool for the non-invasive assessment of myocardial inflammation [24]. Acute inflammatory cardiomyopathies are discussed in the following sections:

### Acute myocarditis

Myocarditis is an inflammatory disease of the myocardium which accounts for 12% of all sudden cardiac deaths on post-mortem studies [25]. Spontaneous recovery of left ventricular function occurs in two thirds of patients but progressive left ventricular dilatation and systolic dysfunction leading to dilated cardiomyopathy (DCM) occur in the remainder [26]. In the acute setting, CMR is established as the imaging tool of choice capable of assessing the distribution, nature and severity of myocardial disease including (i) interstitial oedema, (ii) hyperaemia and inflammatory infiltration and (iii) myocyte necrosis and replacement fibrosis [27]. These features form the CMR Lake Louise Criteria (LLC, Fig. 1) with a diagnostic accuracy of 78% (sensitivity 67%, specificity 91%) when at least two out of three features are present [28]. However, these criteria represent qualitative variables that may be present or absent, guided by a threshold enhancement ratio of ≥2 of myocardium relative to skeletal muscle for interstitial oedema and ≥4 for hyperaemia. Endomyocardial biopsy represents the gold standard tool for the assessment of acute myocarditis [29]. However, this invasive procedure confers a small but tangible risk and is further limited by sampling error due to the focal nature of inflammatory infiltrates [30]. Endomyocardial biopsy performed in a biventricular manner has the greatest yield but is still only ‘positive’ in ∼70% of patients [31] meeting diagnostic criteria for myocarditis by current guidelines [32].

**Fig. 4:**
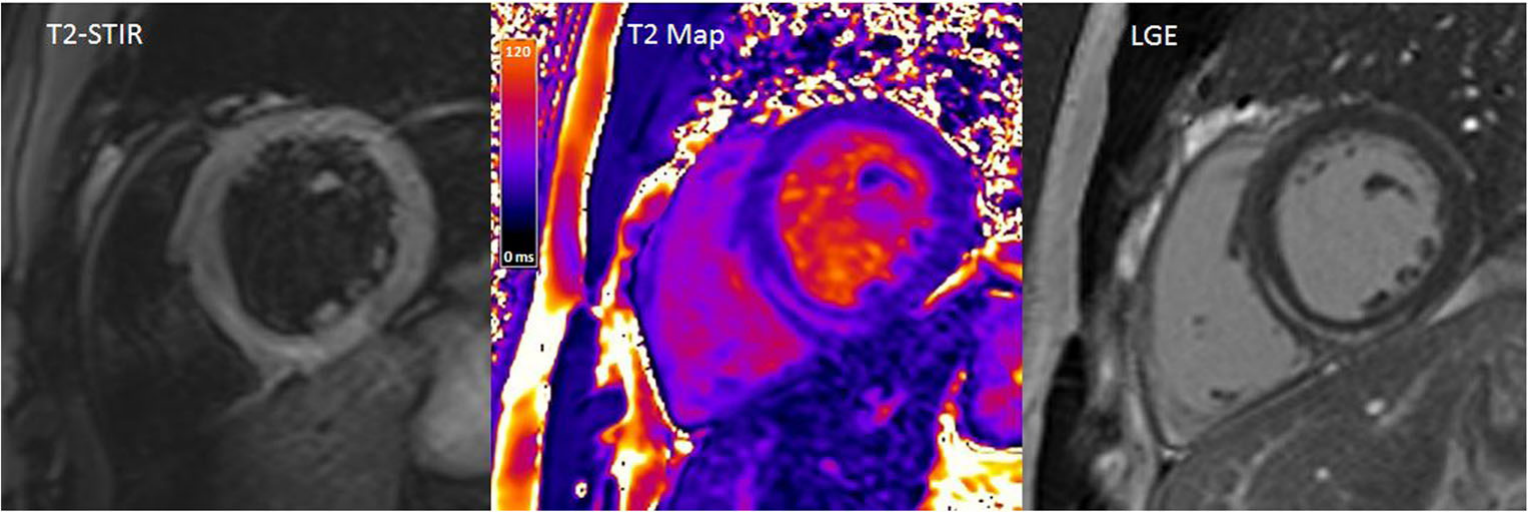
T2-STIR and T2 mapping at the basal short-axis level in a patient with acute myocarditis affecting the inferoseptal wall. Some caution would be required in cardiac walls adjoining the lung, particularly the inferolateral wall, due to B0 distortion effects in some types of sequence, particularly at 3-T field strengths. The late gadolinium enhancement image is provided for reference

Whilst acute inflammatory cardiomyopathies, such as myocarditis, may be assessed by conventional T2-STIR sequences, quantitative mapping techniques allow greater delineation and may reveal myocardial injury not seen on conventional imaging sequences (Fig. 4). T2 mapping was found to reliably detect myocardial involvement extending beyond areas identified by T2-STIR and late gadolinium enhancement with a threshold cut-off value of >59 ms in 30 patients with clinically suspected acute myocarditis [12] and >60 ms in 16 patients with biopsy-confirmed acute myocarditis [33]. The diagnostic performance of T2 mapping was comprehensively studied in a prospective cohort of 129 patients with biopsy-proven acute myocarditis [34]. Diagnostic accuracy with T2 mapping was found to be 81%, which was superior to standard LLC (56%), and this remained true both in the acute and chronic (>14 days) settings. Further studies are underway, including new methods for segmental analysis and mean absolute pixel standard deviation as a measure of tissue inhomogeneity [35], with an emphasis on defining the prognostic significance of elevated T2 values on functional recovery and clinical outcomes.

### Myocarditis vs idiopathic dilated cardiomyopathy

Patients with myocarditis often present with recent-onset heart failure with ongoing low-level myocardial inflammation [32]. In these patients, considerable overlap exists in terms of presentation with idiopathic DCM. In a study of patients with recent-onset heart failure (median time interval of 27 days from symptom onset to presentation), those with biopsy-confirmed active myocarditis had significantly elevated mean global myocardial T2 values compared to those with normal biopsies [33]. There were no other significant differences between groups in terms of baseline demographics, LV ejection fraction (mean 30%), troponin and BNP levels. A global myocardial T2 value of ≥60 ms was also suggested as the optimal cut-off threshold for active myocarditis, consistent with previous studies [12, 33, 34]. From these observations, a global myocardial T2 value of <59–60 ms at 1.5 T may be used to rule out active myocarditis and serve as the gatekeeper for stratifying patients above this threshold for endomyocardial biopsy, with the aim of not only confirming the diagnosis but also establishing underlying aetiology. However, this is subject to calibration and sequence set-up, of which there is no current standard.

### Sarcoidosis

Sarcoidosis is a multisystem, granulomatous disease that most commonly affects young adults with cardiac involvement representing the second most common cause of death. Whilst late enhancement readily detects non-viable myocardial tissue [36], the detection of active myocardial inflammation by T2-STIR faces similar challenges described in earlier sections. As a result, most cases of cardiac sarcoidosis continue to be detected for the first time on post-mortem examination [37]. However, unlike myocarditis, a strong evidence base supports the early use of steroids and other immunosuppressive agents to reverse active inflammation and prevent further deterioration in cardiac function and scar formation [38, 39]. Fluorine-18 fluorodeoxyglucose positron emission tomography computed tomography ([18F] FDG-PET CT) has been explored as an alternative imaging modality, but systematic comparisons have shown that CMR correlates better with clinical disease manifestations [40] and has greater specificity [41].

T2 mapping has been studied in a single retrospective review of 50 consecutive patients with histologically confirmed sarcoidosis undergoing CMR [42]. Amongst patients with suspected cardiac involvement from clinically relevant electrocardiographic and electrophysiological abnormalities, global T2 values were significantly elevated; moreover, 41% of patients showed elevated T2 despite showing no evidence of late gadolinium enhancement. Maximum myocardial T2 exceeding 59 ms was taken as the cut-off, as previously reported in myocarditis [12]. Further studies are required in this area, including head-to-head comparison with hybrid PET-MR-based approaches, which have also been shown to improve sensitivity [43]. However, this technique remains limited by radiation exposure in young individuals, particularly relevant for serial examination to monitor inflammation in response to steroid therapy.

### Myocardial infarction

Acute myocardial ischaemia and infarction are associated with myocardial oedema, although the former represents a potentially reversible injury. For this reason, there has been much interest in the role of T2-STIR to define the areas at risk following acute myocardial infarction [44] and severe transient ischaemia prior to troponin elevation and detection of late gadolinium enhancement [45]. Whilst these observations have important clinical implications, the inherent limitations and unreliability in image quality of T2-STIR imaging has limited widespread use [46]. T2 mapping overcomes many of these limitations, particularly bright signal from low flow of blood adjacent to the subendocardium. In a study of 22 dogs that underwent coronary occlusion followed by reperfusion before CMR, T2 mapping effectively distinguished infarcted myocardium from salvaged myocardium, both of which exhibited T2 values significantly greater than remote myocardium [47]. Whilst further investigation is required to unravel the area at risk, and its dynamic nature, there is evidence that greater T2 values are associated with adverse outcomes following myocardial infarction, which has not been shown in myocarditis or sarcoidosis [48].

Microvascular obstruction (MVO) is known to represent an important clinical predictor of major adverse cardiac events following acute myocardial infarction [49]. Intramyocardial haemorrhage (IMH) occurs in severe forms of MVO and detection may have additional prognostic value [50]. However, accurate differentiation of MVO and IMH by T2-weighted imaging is challenging due the competing effects of haemorrhage (shortens T2—hypointense) and oedema (lengthens T2—hyperintense), combined with low proton density (hypointense) in MVO without IMH [51]. In this setting, T2* imaging was generally found to be more sensitive to haemorrhagic by-products [52].

### Takotsubo cardiomyopathy

Takotsubo syndrome is an acute reversible heart failure syndrome with various anatomical variants and underlying pathophysiological mechanisms [53]. Myocardial oedema and elevated T2 signal intensity have been reported in the acute setting [54]. To date, a single preliminary study has demonstrated that patients with Takotsubo cardiomyopathy had significantly higher T2 values (65 ± 6 ms) compared to healthy controls and that T2 values were significantly higher in segments with wall motion abnormalities compared to normokinetic segments [55]. Further studies are required to elucidate the diagnostic and prognostic significance of these findings.

### Cardiac transplant rejection

Cardiac inflammation in the setting of cardiac transplant rejection represents a diffuse process. One of the strengths of T2 mapping is the ability to identify prolonged T2 relaxation times in the absence of comparison with normal remote myocardium. Early studies have confirmed the positive correlation between prolonged T2 times and biopsy-determined grades of acute transplant rejection, but these approaches were limited by older sequence techniques based on spin echo at 0.5 T [56]. There has been growing interest in the use of T2 mapping to guide whether (i) an endomyocardial biopsy is required to detect transplant rejection and (ii) to monitor treatment response with normalisation of an initially elevated T2 value [57]. A pilot study of 53 transplant patients using updated imaging sequences based on steady-state free precession demonstrated that T2 values were significantly elevated at grade 2R and 3R rejection amongst the 8 patients with biopsy-confirmed rejection [58]. Additionally, these elevated T2 values (mean 60.1 ± 2.1 ms) normalised in all patients when re-scanned after immunosuppression therapy at 2.5 months. A multicentre study is currently in progress to assess if T2 mapping can effectively guide selective use of endomyocardial biopsy in the setting of transplant rejection [59].

### Emerging clinical applications

Cardiotoxicity from chemotherapy represents an important cause of morbidity and mortality amongst patients with cancer. Surveillance of LV ejection fraction during treatment is recommended but represents a late manifestation of cardiac disease, as in the case of iron overload. T2 mapping may offer a clinical tool with higher sensitivity than T2-STIR to detect and quantify subclinical cardiac disease secondary to chemotherapy use. A single study of nine patients receiving anthracycline-based chemotherapy showed that T2 values were elevated amongst those with cardiac disease detected by multigated acquisition scans [60]. Further investigation is required, but T2 mapping in this setting may also represent an important clinical tool to detect early cardiac involvement and to monitor treatment response.

## Future directions

In addition to exploring the role of T2 mapping across a range of cardiovascular diseases previously studied by T2-STIR, there is a major need to focus on defining normal ranges to evaluate the clinical impact of a regional or global T2 value within an individual patient. At present, various T2 mapping sequences are used across multiple vender-specific platforms. Whilst statistical analyses may demonstrate differences between patient groups and contribute to understanding disease processes and responses, there is unmet need to define the significance of a T2 value within a single patient to ultimately guide clinical management. This is exemplified by a similar evolution that occurred in T2* imaging.

## Conclusion

Elevated T2 signal representing oedematous myocardium may be assessed qualitatively by conventional T2-STIR imaging or quantitatively by T2 mapping, for which clinical evidence is emerging across the range of acute inflammatory cardiomyopathies. T2* signal arises from changes in tissue iron level and now serves as a routine clinical tool for assessing iron loading in myocardial and hepatic tissue. Whilst further outcome-based studies are required, T2 mapping is likely to impact routine clinical evaluation of patients with recent-onset heart failure given the ability to detect reversible myocardial inflammation.
